# On the Discovery of Monocular Rivalry by Tscherning in 1898: Translation and Review

**DOI:** 10.1177/2041669517743523

**Published:** 2017-11-29

**Authors:** Robert P. O’Shea, Urte Roeber, Nicholas J. Wade

**Affiliations:** Department of Psychology, School of Psychology and Exercise Science, 5673 Murdoch University, Perth, Australia; Discipline of Psychology, School of Health and Human Sciences, Southern Cross University, Coffs Harbour, Australia; Department of Psychology, School of Psychology and Exercise Science, 5673 Murdoch University, Perth, Australia; Institute for Psychology, University of Leipzig, Leipzig, Germany; Psychology, University of Dundee, Dundee, UK

**Keywords:** perception, rivalry/bistability, history, translation, Tscherning, Breese, Helmholtz

## Abstract

Monocular rivalry was named by Breese in 1899. He made prolonged observation of superimposed orthogonal gratings; they fluctuated in clarity with either one or the other grating occasionally being visible alone. A year earlier, Tscherning observed similar fluctuations with a grid of vertical and horizontal lines and with other stimuli; we draw attention to his prior account. Monocular rivalry has since been shown to occur with a wide variety of superimposed patterns with several independent rediscoveries of it. We also argue that Helmholtz described some phenomenon other than monocular rivalry in 1867.

## Introduction

**Figure 1.**
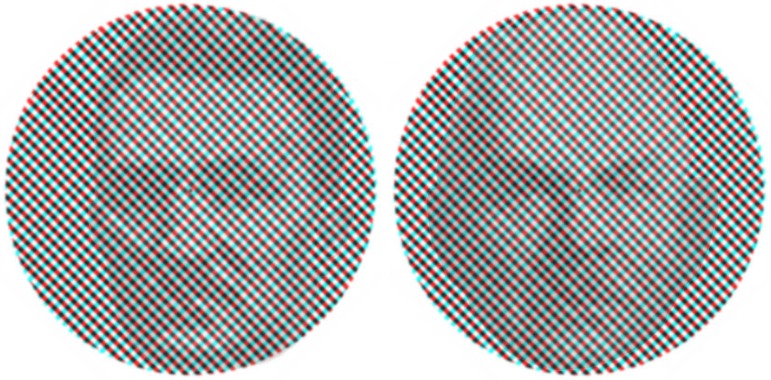
*Monocular rivalries* by Nicholas Wade. Superimposed orthogonal red and green gratings combined with portraits of Marius Hans Erik Tscherning on the left and Burtis Burr Breese on the right. If one fixates steadily the center of either image for at least 30 s, initially the two gratings appear superimposed, but then the clarity (the perceived contrast) of one orientation diminishes, while the clarity of the other enhances for a second or so, and then the reverse. Occasionally, one grating alone will be visible. More frequently, combinations of the gratings are seen in which, for example, the red lines are clearer than the green. Transitions among the various states can also be piecemeal and dynamic.

If we look steadily and patiently at the superimposition of two different images, such as those shown in Figure 1, we will see complementary parts of the images, or all of them, alternate in clarity, if not visibility. Burtis Burr Breese (1868–1939; Figure 1 right) called this *monocular rivalry* (Breese, 1899, p. 42) to contrast it with binocular rivalry in which each component image is presented to a different eye and on which he conducted a long series of studies. One year earlier, Marius Hans Erik Tscherning (1854–1939; Figure 1 left) described similar observations when viewing a grid of vertical and horizontal lines (Tscherning, 1898); he noted the pattern alternations in the course of studying Troxler fading.

Breese (1899) is usually credited with the discovery of monocular rivalry (O’Shea, 1998; O’Shea, Parker, La Rooy, & Alais, 2009; van Boxtel, Knapen, Erkelens, & van Ee, 2008; Wade, 1977) although Tong (2001) suggested that the phenomenon was described by Helmholtz (1867a). We next give the evidence for Tscherning’s primacy, we argue that Helmholtz was describing some other phenomenon, and we add independent accounts of monocular rivalry from Breese (1899), McDougall (1901, 1906), Honisett and Oldfield (1961), Crovitz and Lockhead (1967), Campbell and Howell (1972), and Sindermann and Lüddeke (1972). We begin, however, with a brief review of the phenomenon.

## Why Is Monocular Rivalry Important?

Monocular rivalry occurs between any superimposed images. They can be simple, such as gratings (Atkinson, Fiorentini, Campbell, & Maffei, 1973; Campbell, Gilinsky, Howell, Riggs, & Atkinson, 1973; Kitterle, Kaye, & Nixon, 1974; O’Shea, 1998; Wade, 1975), lines (Andrews & Purves, 1997), or bars (Sindermann & Lüddeke, 1972). Images can be complex, such as of faces and houses (O’Shea et al., 2009). The images can be achromatic, but the phenomenon is more pronounced if the component images differ in color (Wade, 1975). Durations of episodes of greater clarity or visibility of one and the other images are stochastic (O’Shea et al., 2009).

A critical issue for theory is whether the brain processes yielding monocular rivalry are similar to those yielding other multistable phenomena such as reversals of the Necker cube and alternations of visibility during binocular rivalry (Alais & Blake, 2005). If they are then monocular rivalry represents a different way of tapping into a general process in the brain for resolving ambiguity (e.g., Logothetis, Leopold, & Sheinberg, 2003). If the processes differ, then monocular rivalry is of interest in its own right as an unexplained phenomenon. Evidence for the former theory comes from at least three lines of evidence:

1. Monocular rivalry and binocular rivalry alternations in adjacent areas of the visual field synchronize with each other (Andrews & Purves, 1997; Pearson & Clifford, 2005).
2. Monocular rivalry shows several of the hallmarks of binocular rivalry, including that suppression yields a reduction in visual sensitivity (O’Shea et al., 2009) and that rivalry increases as differences between the rivalry stimuli, such as color, increase (Knapen, Kanai, Brascamp, van Boxtel, & van Ee, 2007; O’Shea, 1998).
3. Quantitative differences between monocular rivalry and binocular rivalry disappear when displays are alternated at various rates with blank fields (van Boxtel et al., 2008).

The weight of evidence suggests that monocular rivalry is explained by its being another example of a general mechanism for dealing with ambiguous inputs.

## Tscherning on Monocular Rivalry

Tscherning was a student of Peter Ludvig Panum in Copenhagen before going on to train in ophthalmology. Panum was an authority on binocular vision including rivalry (Panum, 1858). Tscherning’s interests in myopia overlapped significantly with those of Louis Émile Javal who invited Tscherning to visit him at the Sorbonne. Tscherning took up the position of Javal’s assistant and remained at the Sorbonne for the next 26 years, where he became director of the ophthalmological laboratory when Javal retired because of the onset of his blindness from glaucoma. Whereas Javal had translated Helmholtz’s *Handbuch* into French (Helmholtz, 1867b), Tscherning translated and annotated Thomas Young’s papers on optics and the eye (Young, 1894).

In his *Optique physiologique* (Tscherning, 1898, 1900), he covered topics such as the optics of light and lenses, dioptrics, optical aberrations, entoptical phenomena, accommodation, ophthalmoscopy, and the psychophysics of light and of color. In Chapter 18 on “The Form Sense” the final section was concerned with Troxler’s phenomenon—the disappearance and reappearance of stimuli away from a fixation point (Devyatko, Appelbaum, & Mitroff, 2017; Troxler, 1804). Tscherning wrote:^1^

**Figure 2.**
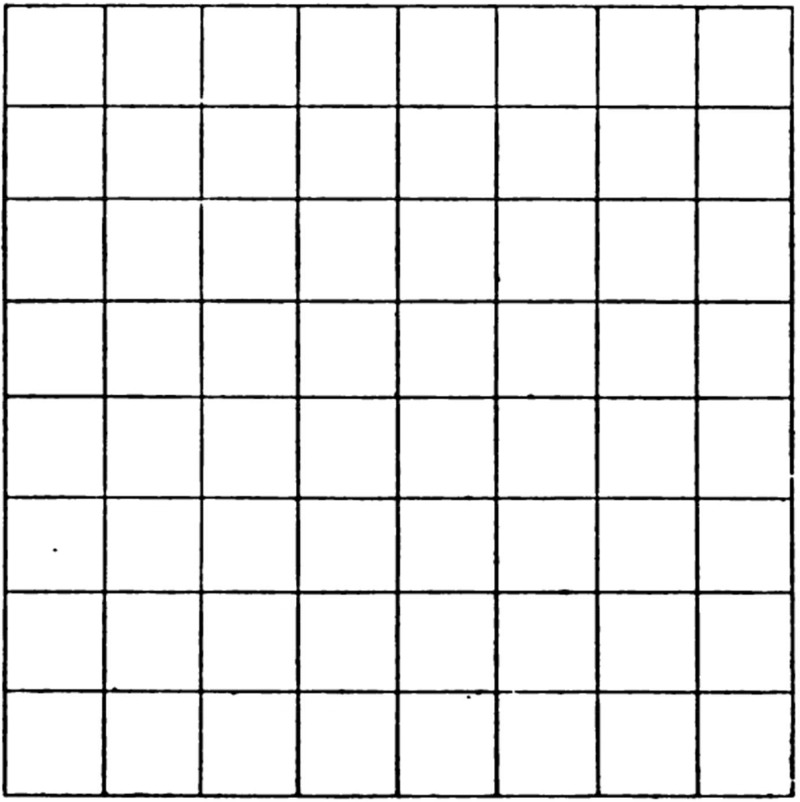
Tscherning’s (1898) stimulus for observing monocular rivalry; his Figure 174.

To study the phenomenon, we can observe a regular drawing, for example, that of figure 174 [Figure 2]. For my eye, the phenomenon begins after fixing the image for eight or ten seconds, that is to say at the moment when fixation begins to be less sure. From this moment, the figure shows perpetual changes: sometimes part of the figure disappears, sometimes another. What is curious is that most often the scotomata are not absolute: sometimes the horizontal lines disappear in one place, while the vertical lines persist, sometimes the opposite takes place. These phenomena are very reminiscent of what has been described as the antagonism of visual fields [binocular rivalry], and is observed, for example, by presenting, in a stereoscope, horizontal lines to one eye, and vertical lines to another. (Tscherning, 1898, pp. 264–265)

**Figure 3.**
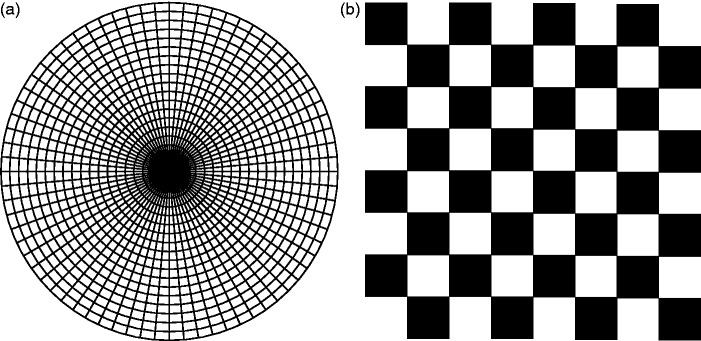
Illustration of patterns like Tscherning’s other stimuli for observing monocular rivalry. A. Concentric circles and radial lines. B. Checkerboard.

Tscherning also noted that the pattern fluctuations occur with other stimuli although he did not illustrate them: “If we fix the center of a figure composed of concentric circles and rays, we see now the latter, sometimes the circles. On a checkerboard one sees sometimes one, sometimes another one of the checks disappear, and so on” (1898, p. 265). The stimuli were probably like those shown in Figure 3.

Tscherning’s depicted stimulus (Figure 2), his description of his experiences when looking at it, and his implicit theory are all similar to those of others who later investigated monocular rivalry. For example, Crovitz and Lockhead (1967) used a similar grid (except rotated by 45°), and reported similar experiences. They too were struck by the similarities of the perceptual experiences to that of binocular rivalry between the two component sets of lines viewed one to each eye.

The evidence for Tscherning’s being the discoverer of monocular rivalry comes from the publication date of his book—1898—one year prior to the independent discovery of the phenomenon by Breese. Breese retains primacy in calling the phenomenon monocular rivalry. This name has stuck in spite of two attempts to change it (Campbell & Howell, 1972; Maier, Logothetis, & Leopold, 2005).

## Breese on Monocular Rivalry

Breese studied at Harvard before taking a fellowship at Columbia, where he published his doctoral dissertation “On inhibition” (Breese, 1899) which represented one of the first quantitative studies of binocular rivalry. Following further research in Europe, he was appointed to chairs in psychology at Tennesse and Cincinnati where he published his textbook on psychology (Breese, 1917). The section on monocular rivalry in Breese (1899) came at the end of his experimental studies on binocular rivalry and he used the same paired stimuli—orthogonal, oblique green, and red gratings on a black background. These were superimposed using either a prism or by transmission and reflection from a glass plate: “The apparatus for Ragona Seinà’s [sic] experiment of mirror contrasts serves as a convenient means for superimposing the fields” (1899, p. 44). Breese either made an error or there was a misprint because the method was that of Ragona-Scinà (O’Shea, Brini, & Wade, 2016). Breese described monocular rivalry in the following way:

Now the interesting part of the experiment is that if the center of the field was fixated, a rivalry of colours was perceptible. Neither disappeared entirely: but at times the red would appear very distinctly while the green would fade; then the red would fade and the green appear distinctly. The two sets of lines showed the same fluctuation, keeping pace with the changing intensities of the colors. Sometimes one of them would disappear altogether.

The rivalry of the colors and of the lines was much slower than the rivalry in binocular vision. A very slight movement of the eye to the right or left would cause changes in the intensities of the fields [because of technical limitations from Breese’s method of superimposing the images]; so that extreme care was necessary in order to avoid the movements, or, at least, not to confuse changes caused by them with the rivalry of the fields, which is independent of eye movements. (1899, pp. 43–44)

Breese did not apply the systematic measurements of predominance durations and alternation frequencies to monocular rivalry as he had to binocular rivalry, possibly because of the evanescent nature of the former. The theoretical conclusion that Breese reached was: “Binocular rivalry, then, would be at once ‘psychical’ and ‘physiological’ in that it is dependent upon central processes, and is affected by the nature of the motor adaptations” (1899, p. 48). He was reflecting on the debate that separated Helmholtz, who was in the *psychical* camp, from Panum and Hering whose interpretations were more *physiological* (see Tong, 2001; Wade & Ngo, 2013).

## Helmholtz on Monocular Rivalry?

Helmholtz had written extensively on binocular rivalry; Tong (2001) has suggested that he described monocular rivalry, too: “Helmholtz discovered that a similar but weaker form of perceptual conflict can occur when two objects are optically superimposed and presented to the same eye, a phenomenon later termed ‘monocular rivalry’ by Breese (1899, p. 57).”

Helmholtz wrote:

Something like this conflict [binocular color rivalry], only much less pronounced, may be also noticed in the monocular field by using an unsilvered plate of glass to reflect the image of an object at the same place where another object as seen through the glass happens to be. The two images in this case should both be equally bright and well defined, but entirely different in pattern. Then we may look at either of the two, and the other one will retire more or less out of sight, although it may never disappear completely, as it does when the images are binocularly superposed*.* (1925, p. 511)

Helmholtz’s description does seem plausible. He used a glass plate to achieve the optical superimposition necessary for monocular rivalry, adopting the approach of Osann (1833; see O’Shea, Roeber, & Wade, 2017, for a translation) and of Ragona-Scinà (1847; see O’Shea et al., 2016, for a translation). He found the alternations to be less pronounced than for binocular rivalry, which is true (Breese, 1899; Wade, 1975). He also attributed the fluctuations in clarity to attention, as he applied to binocular contour rivalry. However, we argue that this description does not meet the standards required to award discovery to Helmholtz for five reasons.

1. **Helmholtz contradicted himself about monocular rivalry**. Helmholtz contrasted perception of a flat image of a grid of oblique lines, a similar stimulus to that used by Tscherning for showing monocular rivalry, with when the two sets of lines were delivered to opposite eyes, yielding binocular contour rivalry. After arguing that binocular rivalry shows
  that the content of each separate field [eye] comes to consciousness without being fused with that of the other field by means of organic mechanisms; and that, therefore, the fusion of the two fields in one common image, when it does occur, is a psychic act. (Helmholtz, 1925, p. 499)

**Figure 4.**
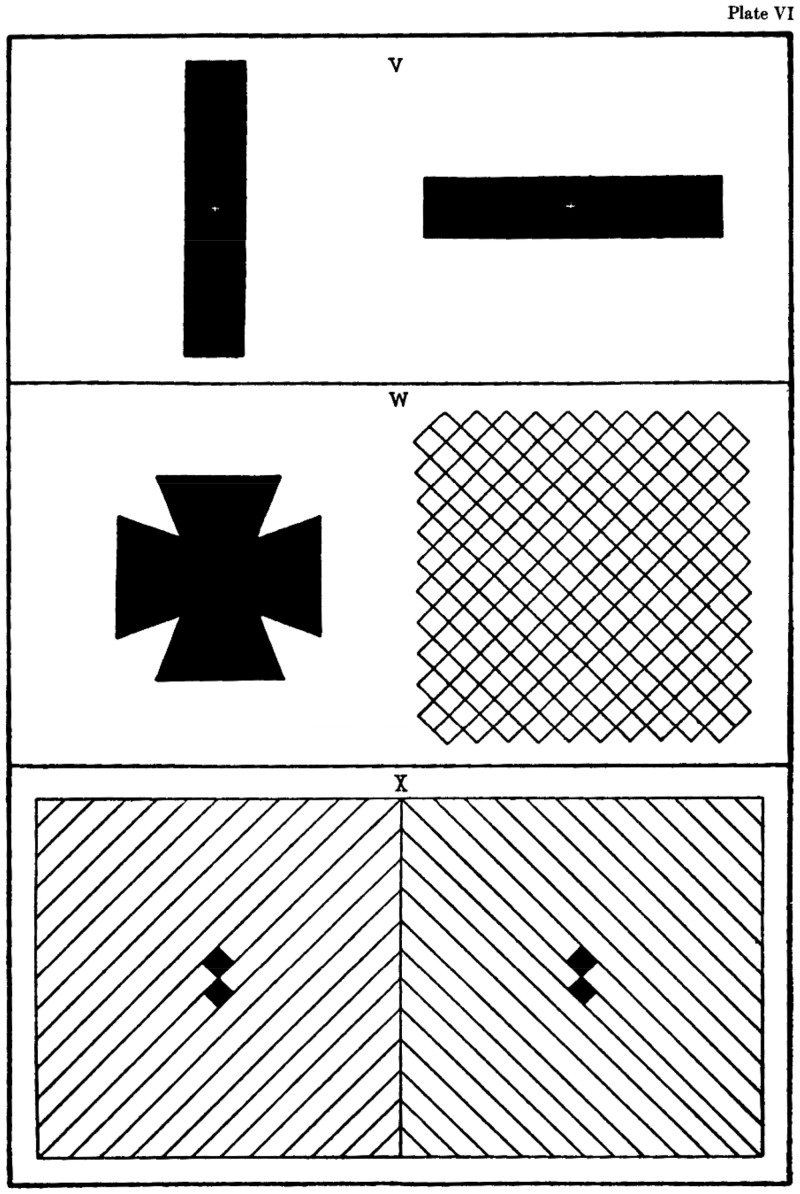
Helmholtz’s Plate XI showing three stereograms for binocular rivalry. Panel V shows a vertical bar to the left eye and a horizontal bar to the right eye. Panel W shows a Maltese cross to the left eye and an oblique grid to the right eye. On looking with the right eye only, Helmholtz said he saw no changes in the visibility of the lines. Panel X shows a set of right oblique lines to the left eye and a set of left-oblique lines to the right eye that yields clear changes in the visibility of the two sets of lines—binocular rivalry.

Helmholtz wrote:

To emphasise this difference [between organic and psychic fusion], we need only compare the binocular fusion of both the sets of oblique and differently oriented lines in Fig X [Figure 4], Plate XI with the monocular union of both in the set of lines in Fig W. In the latter we also can count the lines of one system or compare their distances [between the lines] but the lines of *the other set will never disappear* from the picture as it does usually with binocular fusion [rivalry]. Under monocular viewing of the combined line set in Fig. W, we have only *one sensory impression that we cannot change with any attentional effort* even though we might pay attention to the one or the other kind [orientation] preferentially. If both line sets of Fig X [under binocular rivalry] were to combine into one single sensory impression then this impression could in no way be separated into its components by attentional effort alone. It is also significant that if one monocularly superimposes the image of the bright sky with a printed page using an unsilvered glass plate then with certain intensities one cannot read the letters while one can read them very well if one binocularly superimposes a much stronger reflection from a mirror. (Helmholtz, 1867a, p. 781, our emphases)

This section is difficult to reconcile with the interpretation that Helmholtz discovered monocular rivalry as we explain later.

1. **Helmholtz was incorrect in saying that contours of one set of lines never disappear in favor of the other set during monocular rivalry**. They occasionally disappear but much less frequently than in binocular rivalry (Wade, 1975). Tscherning also reported disappearances, as have all other researchers.

Our own observations on looking steadily with both eyes at the right half image of Helmholtz’s stereogram W (Figure 4, middle panel) are that we see lots of perceptual changes taking place including monocular rivalry. We see numerous brief changes in perceptual organization, such as from vertical to horizontal strings of diamonds, to different brooches of adjacent diamonds, and to large Xs, Us, and rectangles made up of many adjacent diamonds. McDougall (1906) reported similar observations in a similar stimulus; we call these changes in perceptual organization (e.g., Attneave, 1971) to distinguish them from monocular rivalry. McDougall reported monocular rivalry in the same stimulus viewed as an afterimage.

Occasionally, we see monocular rivalry: The left-oblique lines are clearer than the right-oblique lines and then we see the reverse. Fleetingly, we do see disappearances of one or the other set of component lines that Tscherning reported with his grid. Our observations of disappearances agree also with Honnisett and Oldfield (1961) as well as with Crovitz and Lockhead (1967) who used an almost identical, black-and-white, oblique grid. Yet Helmholtz did not report any of these observations.

1. **There is an alternative interpretation of Helmholtz’s words.** To reconcile why different observers might have seen binocular mixing of dichoptically presented colors, whereas he saw rivalry, Helmholtz made the remarkable claim that when he optically superimposed a patch of blue and a patch of red to one eye, although it appeared violet, he could nevertheless see the component colors: “when colours are mixed monocularly, there are certain cases when we *imagine* we see one of the combined colours through the other…. [I]t happens whenever we are induced to separate a coloured illumination or mantle from some coloured object” (Helmholtz, 1925, p. 510, our emphasis)

It is telling that Southall translated Helmholtz’s (1867a) “zu sehen glauben” (p. 781) as “imagine”—there is no sense of any change in clarity or visibility characteristic of monocular rivalry in Helmholtz’s words. We should mention that there was considerable dispute in Helmholtz’s time about whether binocular color rivalry exists or whether dichoptically presented colors mix and fuse (Howard & Rogers, 2012, pp. 325–327). Helmholtz equated binocular color rivalry with the monocular mixing in the above quotation, but nowadays these are regarded as quite different phenomena.

When we look at two transparent colored surfaces that partially overlap, simulating what happens when an afterimage is projected onto a colored surface, we too can imagine that we can see both colors in the region of overlap, but the color there is governed by laws of transparency (e.g., Ekroll & Faul, 2013) and does not alternate in clarity or visibility. In both of these examples, Helmholtz’s claim of being able to see both colors is not an example of monocular color rivalry.

1. **There is a possible optical explanation for Helmholtz’s report.** How do we reconcile Helmholtz’s failure to see monocular rivalry in a stimulus similar to that from which others see monocular rivalry with his reports of fluctuations in clarity of a reflection of an object and direct view of a different real object? Unless Helmholtz was careful to equalize the viewing distance of the two images, then a simple explanation of one object’s “retir[ing] more or less out of sight” (p. 511) when he was looking at the other is *accommodative blur*. That is, when looking at one object, another object at a different viewing distance will be blurred by the limited depth of field of the eye. If so, it is not surprising that a blurry image is less visible than a sharply focused one, or that this can switch to the other object when Helmholtz looked at it. But this is to violate one of the conditions for monocular rivalry—that it occurs without any change in the input to the eyes.
2. **Neither Tscherning nor Breese credited Helmholtz with discovering monocular rivalry.** Tscherning and Breese were familiar with Helmholtz’s *Handbuch.* Tscherning’s (1898) name index lists 62 pages on which Helmholtz is mentioned. Tscherning also wrote glowingly about Helmholtz’s *Handbuch* in his section on further reading (p. 333), recommending that readers prefer the original German text instead of the translation into French that was made in 1867. Yet, Tscherning did not mention Helmholtz anywhere in his section on Troxler fading and monocular rivalry.

Breese (1899) showed great care in citing work relevant to his review of inhibition and to his experiments in binocular rivalry, including many German texts. Indeed, he said, “Helmholtz was the first investigator who studied binocular rivalry carefully” (p. 18) and he summarized Helmholtz’s theories about, and observations of, binocular rivalry (pp. 18–19). Breese’s careful honoring of Helmholtz’s preceding work on binocular rivalry is inconsistent with his failing to cite Helmholtz as discovering monocular rivalry.

That two careful scholars who clearly read some edition of Helmholtz’s *Handbuch* failed to credit him with discovering monocular rivalry lends credibility to our argument that Helmholtz was describing something other than monocular rivalry.

## Later History of Monocular Rivalry

Prior to our finding the work of Tscherning, and prior to the claim, which we dispute, that Helmholtz discovered monocular rivalry, Breese (1899) was regarded as its discoverer. We argue that Breese discovered monocular rivalry independently. Because Breese did not cite Tscherning (1898), we conclude that he was unaware of it. Moreover, Breese’s approach was very different from Tscherning’s. Breese’s main interest was in binocular rivalry, but he was able to modify his stereoscope so both rival images were delivered to the same eye. This modification required careful fixation—something that appears to be necessary for monocular rivalry to occur.

Thereafter, it is a similar story: Neither Honisett and Oldfield (1961) nor Crovitz and Lockhead (1967) cited Tscherning or Breese, suggesting they discovered monocular rivalry independently. Neither did Sindermann and Lüddeke (1972), also consistent with independent discovery. Finally, Campbell and Howell (1972) were also unaware of Tscherning’s or Breese’s work. They did cite Breese later (e.g., Campbell et al., 1973).

## Conclusion

We have shown that Tscherning (1898) described monocular rivalry in a variety of stimuli, giving him the credit for discovering the phenomenon. We have marshalled five lines of evidence casting doubt on the claim that Helmholtz (1867a) discovered monocular rivalry. We argue that the phenomenon has been independently discovered at least five more times before 1973.

## Supplementary Material

Supplementary material

## Note

1 In the Supplementary Materials, we provide the original French text of the entire section from Tscherning (1898, pp. 264–265) along with a translation into English.
