# Supplementary material for: On the Discovery of Monocular Rivalry by Tscherning in 1898: Translation and Review
Source: Iperception. 2017 Nov 29;8(6):2041669517743523. doi: 10.1177/2041669517743523 (PMC5714086; doi:10.1177/2041669517743523)
Supplement: Supplementary material [file supplementary_material.pdf]

OPTIQUE PHYSIOLOGIQUE

DIOPTRIQUE OCULAIRE — FONCTIONS
DE LA RÉTINE — LES MOUVEMENTS OCULAIRES
ET LA VISION BINOCULAIRE

LEÇONS PROFESSÉES A LA SORBONNE

PAR
LE Dr TSCHERNING
DIRECTEUR-ADJOINT DU LABORATOIRE D'OPHTALMOLOGIE

LANE MEDICAL LIBRARY
8693
SAN FRANCISCO

PARIS
MASSON ET Cie, ÉDITEURS
LIBRAIRES DE L'ACADÉMIE DE MÉDECINE
120, Boulevard Saint-Germain

1898

PHYSIOLOGICAL OPTICS

DIOPTRICS — FUNCTIONS
OF THE RETINA — EYE MOVEMENTS
AND BINOCULAR VISION

———--

PROFESSED LESSONS AT THE SORBONNE

By

DR TSCHERNING
DEPUTY DIRECTOR OF THE LABORATORY OF OPHTHALMOLOGY

PARIS
MASSON AND CO., PUBLISHERS
LIBRARIES OF THE ACADEMY OF MEDICINE
180 Boulevard Saint-Germain

1898

Translated by Robert P. O'Shea, 23 Sep 2017.

Phénomène de Troxler. — Si l'on dessine plusieurs taches noires sur une feuille de papier et qu'on en fixe une pendant quelque temps, on voit tantôt une, tantôt une autre des taches environnantes disparaitre pour réapparaitre peu de temps après, le plus souvent à l'occasion d'un battement des paupières ou d'un petit mouvement de l'ceil. Ce singulier phénomène, qui a été décrit au commencement de ce siècle par *Troxler*,

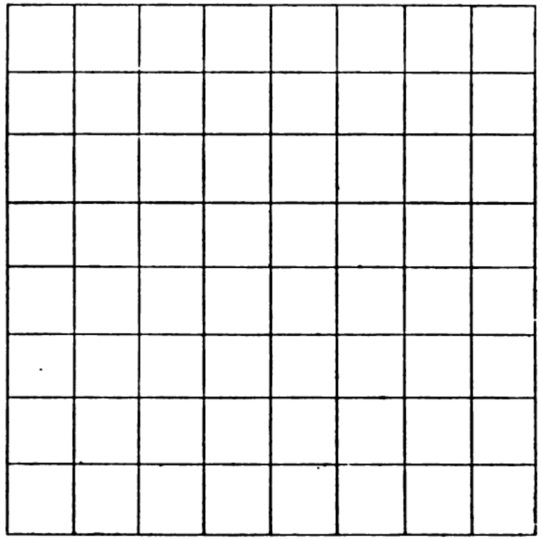

Fig 174

a été étudié dernièrement par je docteur *Holth*. La couleur du fond, ainsi que celle des taches, ne joue aucun rôle: pendant la disparition de ces dernières, on ne voit à leur place que le fond le scotome se remplit done à peu prés comme la tache de *Mariotte*. La tache fixée peut même dinparattre, après un long temps de fixation. Pour étudier le phénoméne, on peut observer un dessin régulier, romane par exemple celui de la figure 174. Pour mon oeil, le phénoméne commence après avoir fixé le milieu pendant huit ou dix secondes, c'est-à-dire au moment où la fixation commence à être moins sûre. Dés ce moment, la figure montre des ehangements perpétuels tantôtune partie de la figure disparait, tantôt une autre. Ce qui est curieux, c'est que le plus souvent les scotomes ne sont pas absolus: tantôt ce sont les lignes horizontales qui disparaissent à un endroit, tandis que les lignes verticales persistent, tantôt

Phenomenon of Troxler. — If one draws several black spots on a sheet of paper and looks fixedly at one for a time, one sees one or another of the surrounding spots disappear and reappear shortly afterwards, more often than not on blinking or on a small movement of the eye. This singular phenomenon, which was described at the beginning of this century by *Troxler*,

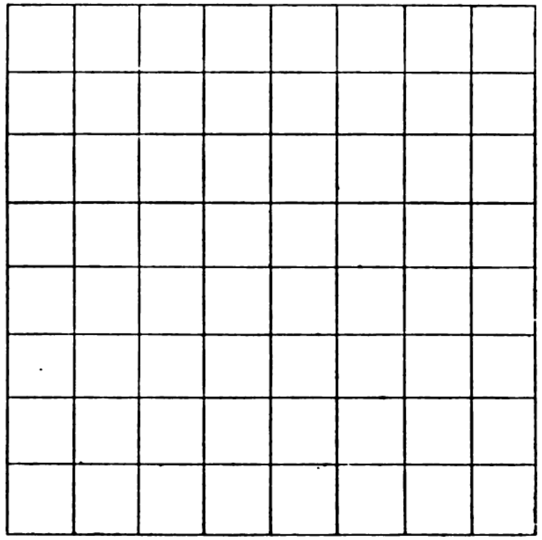

Fig. 174

has lately been studied by Dr. *Holth*. The color of the background, as well as that of the spots, plays no part: during the disappearance of the latter, one sees in their place only the background, the scotoma thus filling almost like the spot of *Mariotte* [the blind spot]. The fixated spot can even disappear after a long time. To study the phenomenon, we can observe a regular drawing, for example, that of figure 174. For my eye, the phenomenon begins after fixing the image for eight or ten seconds, that is to say at the moment when fixation begins to be less sure. From this moment, the figure shows perpetual changes: sometimes part of the figure disappears, sometimes another. What is curious is that most often the scotomata are not absolute: sometimes the horizontal lines disappear in one place, while the vertical lines persist, sometimes

c'est le contraire qui a lieu. Ces phénomènes rappellent beaucoup celui qu'on a décrit sous le nom d'antagonisme des champs visuels et qu'on observe, par exemple, en présentant dans un stéréoscope des lignes horizontales à un oeil, des lignes verticales à un autre. — Si l'on fixe le centre d'une figure composée de cercles concentriques et de rayons, on voit tantôt ces derniers, tantôt les cercles. Sur un damier on voit tantôt une, tantôt une autre des cases disparaitre, et ainsi de suite. *Holth* a méme fait disparaitre des objets lumineux, la lune par exemple; suivant lui, des petits objets disparaissent mente si on leur imprime un mouvement lent. Il y a donc lieu de se méfier de cette source d'erreurs, si l'on désire faire de la périmétrie de précision.

B**ibliographie.** — …. Troxler. *Ueber das Verschwinden gegebener Gegenstände innerhalb unseres Gesichtskreises innerhalb unseres Gesichtskreises. Himly u. Schmidt. Ophthalm Bibliothek.*, 1802, II, p. 1…. — Holth (S.). *Om det normale Synsorgans Stirreblindlied. Norsk Magazin for Laegevidenskaben.* August 1895.

the opposite takes place. These phenomena are very reminiscent of what has been described as the antagonism of visual fields [binocular rivalry], and is observed, for example, by presenting, in a stereoscope, horizontal lines to one eye, and vertical lines to another. If we fix the center of a figure composed of concentric circles and rays, we see now the latter, sometimes the circles. On a checkerboard one sees sometimes one, sometimes another one of the boxes disappear, and so on. *Holth* has even made luminous objects disappear, the moon for example; according to him, small objects disappear if a slow motion is imparted to them. We must therefore beware of this source of errors, if we wish to do perimetry precisely.

B**ibliography.** — [Troxler, I. P. V. (1804). Über das Verschwinden gegebener Gegenstände innerhalb unseres Gesichtskreises [On the disappearance of given objects from our visual field]. *Ophthalmologische Bibliothek, 2*(2), 1-53.] ….— [Holth, S. (1896). Det normale Synsorgans indirekte Stirreblindhed og dens Betydning under Synsfeltundersøgelser [Blindness of normal vision and its significance during visual field studies]. *Norsk magazin for lægevidenskaben*(Supplement), 1-148. Retrieved from http://www.nb.no/nbsok/nb/eb76e82d8dbdac1a09073e3a6b1c2919.nbdigital?lang=no#5]
